# Altered GABA and secondary bile acids in Guillain-Barré syndrome: association with gut dysbiosis

**DOI:** 10.3389/fimmu.2026.1849216

**Published:** 2026-06-10

**Authors:** Jiafang Fu, Jingli Shan, Hua Xu, Zhiwei Zhu, Pengshuo Yang, Qinzhou Wang, Jinxiang Han, Guangxiang Cao

**Affiliations:** 1 Department of Neurology, The First Affiliated Hospital of Shandong First Medical University & Shandong Provincial Qianfoshan Hospital, Jinan, China; 2 Biomedical Sciences College and Shandong Medicinal Biotechnology Centre, Shandong First Medical University and Shandong Academy of Medical Sciences, Jinan, China; 3 National Health Commission (NHC) Key Laboratory of Biotechnology Drugs, Shandong Academy of Medical Sciences, Jinan, China; 4 Research Institute of Neuromuscular and Neurodegenerative Diseases and Department of Neurology, Qilu Hospital, Cheeloo College of Medicine, Shandong University, Jinan, China; 5 Shandong Institute of Industrial Technology for Health Sciences and Precision Medicine, Jinan, China

**Keywords:** gamma-aminobutyric acid, Guillain-Barré syndrome, gut microbiota dysbiosis, metabolic disorder, secondary cholic acid

## Abstract

**Objective:**

Guillain-Barré syndrome (GBS) is a rare, immune-mediated inflammatory disease of the complex peripheral nervous system that often follows acute infections, and may also be associated with long-term ‘silent infections’. Long-term “silent infections” can alter the gut microbiota, which in turn may contribute to immune-mediated inflammatory diseases. Emerging evidence suggests that gut dysbiosis and altered serum metabolites are associated with GBS, but the causative link between GBS and gut microbiota remains unclear. Therefore, this study aimed to evaluate the association between gut microbiota structure and serum metabolic profile in GBS.

**Methods:**

Untargeted metabolomics profiling of serum and metagenomics sequencing of stool samples were performed to capture the global metabolic and microbial differences between GBS subjects and healthy controls. Multivariate statistical analyses, including PLS-DA, were applied to identify distinct clustering patterns and differential abundances of metabolites and gut microbiota. Pearson’s correlation analysis was used to estimate the correlations between abundance of gut microbiota and serum metabolic profile. Seven different media were used to isolate the potential pathogens from GBS stool samples.

**Results:**

The metabolome data revealed that gamma-aminobutyric acid (GABA) metabolism and secondary cholic acid metabolism were perturbed in GBS. Specifically, GABA was increased significantly (approximately 14.3-fold), while multiple secondary cholic acids (methyl deoxycholate, glycodeoxycholic acid, glycolithocholic acid, taurolithocholic acid, and coprocholic acid) were decreased significantly in GBS subjects. Regarding the gut microbiota identified via metagenomic sequencing of stool samples, *Ligilactobacillus salivarius*, *Enterocloster bolteae*, and the opportunistic pathogenic *Klebsiella pneumonia* were notably more abundant in GBS subjects, while *Bacteroides* sp., *Roseburia hominis* and *Paraprevotella xylaniphila* were decreased significantly. In addition, pathogens such as *K. pneumoniae* were also isolated from GBS subjects. Further analysis of the metagenomic data revealed enrichment of prokaryotic genes involved in the GABA biosynthesis pathway, while genes associated with secondary cholic acid metabolism pathways were decreased in gut microbiome in GBS subjects. On this basis, correlation analysis revealed that changes in GABA were associated with altered levels of gut microbes including *Enterococcus* species, *Ligilactobacillus salivarius* and *Enterocloster bolteae*, whereas changes in secondary cholic acids were positively correlated with altered levels of *Bacteroides* species and *Roseburia* species.

**Conclusion:**

GABA metabolism and secondary cholic acid metabolism were significantly disturbed in GBS subjects, potentially resulting from the dysbiosis of the gut microbiota. *K. pneumonia* and other no gut microbes were significantly enriched and isolated in GBS and may contribute to the inflammatory response in this immune-mediated inflammatory disease. These findings also suggest that GABA may be a promising biomarker for the diagnosis of GBS and that modulation of gut microbiota might impact the clinical course of GBS.

## Introduction

Guillain-Barré syndrome (GBS) is a rare, acute idiopathic polyneuropathy that typically develops after a previous gastrointestinal or respiratory infection (e.g., *Campylobacter jejuni*, *Mycoplasma pneumoniae*, *Haemophilus influenzae*, and cytomegalovirus) (1–5). Although GBS is an immune-mediated inflammatory disease of the peripheral nervous system, knowledge of the exact pathogenic factors that activate the immune system in GBS is limited. However, prodromal infection may be the cause of complex neurological diseases, with evidence suggesting that specific microbial infections, such as *Clostridium perfringens* and *herpes simplex virus*, can trigger immune responses that eventually lead to the occurrence of diseases of the nervous system (6, 7). Prodromal infections are recognized as important triggers of GBS (8–10). Epidemiological and serological studies have established that preceding infections with *Campylobacter jejuni*, *Mycoplasma pneumoniae* or cytomegalovirus are commonly associated with the development of GBS (11, 12). Specifically, *C. jejuni* is the most frequently identified pathogen preceding GBS, while *M. pneumoniae* and cytomegalovirus have also been consistently reported as antecedent infectious agents (13). With the progress of gut microbiota research, an increasing number of studies have suggested that long-term dysbiosis (a chronic imbalance of gut microbiota) may be an important factor leading to metabolic disorders, inflammatory reactions and immune responses (14, 15). Accumulating evidence has revealed complex interactions among gut microbiota, host metabolism, and immunity in various inflammatory and autoimmune diseases (15, 16); however, the potential relationships among gut microbiota, metabolic disorders and the immune system remain unknown in GBS.

Characterizing the biomarkers present in the serum of GBS patients and understanding the role of such biomarkers in GBS pathogenesis could aid in the development of more effective GBS treatments. Currently, there are no well-defined biomarkers for GBS. However, several serum-derived markers, such as CD23, IL-1α and IL-9, have been identified as potential biomarkers in serum samples from GBS patients (2), and the neurofilament light chain may be a potential biomarker for axonal damage in GBS (17).

GABA is the most important inhibitory neurotransmitter in the central nervous system of mammals, playing a critical role in maintaining the excitation-inhibition balance and regulating neural circuit functions (18). In addition, GABA also modulates peripheral nerve function and immune responses. In the peripheral nervous system, local GABAergic signaling regulates nociceptive transmission and pain (19), and the GABA analogue pregabalin is a first−line treatment for diabetic neuropathic pain (20). GABA exerts immunomodulatory effects on T cells and antigen−presenting cells, and alters disease severity in autoimmune models such as experimental autoimmune encephalomyelitis (21, 22). GABA can be synthesized not only by the host but also by gut microbiota, such as those belonging to the genera *Enterococcus* and *Bifidobacterium* (23, 24). Gut microbiota−derived secondary bile acids also play an important role in the pathogenesis of autoimmune diseases. Secondary bile acids serve as key signaling molecules linking the gut microbiota to the regulation of immune system homeostasis (25). In patients with multiple sclerosis (MS), the abundance of gut microbes responsible for secondary bile acid production is significantly reduced, leading to decreased deoxycholic acid levels and an increased proportion of Th17 cells (26). However, the roles of GABA and secondary bile acids in GBS remain unclear.

In a previous study by our research group, untargeted metabolomics and metagenomics analysis revealed that the levels of certain gut microbes, such as *Bacteroides ovatus*, *Bacteroides caccae* and *Ruminococcus gnavus*, correlated with the metabolism of bile acids and arachidonic acid in patients with chronic inflammatory demyelinating polyradiculoneuropathy (CIDP) (27), which is also an immune-mediated inflammatory disease of the peripheral nervous system, revealing that gut microbiota may affect the occurrence and development of CIDP through the gut-organ axis. Although both GBS and CIDP are immune−mediated peripheral neuropathies, their clinical courses and pathogenic mechanisms differ. In addition, previous studies have focused on the association between preceding microbial infections and GBS, whereas no research has addressed the relationship between gut microbiota and GBS. Therefore, in this study, we sought to investigate whether similar gut−metabolite alterations exist in GBS and to identify GBS−specific changes by comparing with CIDP. We performed untargeted metabolomics profiling of serum to discover potential biomarkers for GBS and conducted a metagenomics analysis of stool samples to discover the association between serum metabolites and gut microbiota in GBS. Here we show that gut microbiota dysbiosis is associated with altered GABA and secondary bile acid metabolism in GBS.

## Methods

### Study population and sample handling

This study enrolled 60 subjects at Qilu Hospital of Shandong University from March 2021 to April 2023, including 30 GBS patients and 30 age and sex matched healthy controls (**Supplementary Table 1**). Inclusion criteria included: (1) The diagnosis of GBS was based on established guidelines as previously described (28); (2) age ≥18 years; (3) disease onset within 2 weeks prior to sample collection; (4) The absence of any severe systemic infection (including but not limited to bacterial meningitis, tuberculosis, severe pneumonia, and septicemia) within the 14−day period preceding blood collection. Exclusion criteria included: (1) Any condition impairing brain function (e.g., stroke, encephalitis, subarachnoid hemorrhage); (2) gastrointestinal diseases (e.g., inflammatory bowel disease, irritable bowel syndrome); (3) use of antibiotics, probiotics, or prebiotics within 4 weeks before sampling; (4) Any serious disorder of other organs (e.g., systemic infection, malignancy, hepatic or renal insufficiency, hematological disease); (5) Incomplete clinical documentation. Thirty healthy controls, who were age- and sex-matched to the patients, were recruited from the health management center of the same hospital. Serum and fecal samples of the subjects were collected and treated as previously described (27). The study was reviewed and approved by the Ethics Committee of Qilu Hospital of Shandong University (ref. 2020047).

### Metabolite extraction

Metabolites were extracted from frozen serum as previously described (29, 30) with modifications. For the extraction, precooled extractant containing methanol:acetonitrile:water at a ratio of 4:2:1 (v/v/v) was added. The mixture was then vortexed for 1 minute and incubated at -20 °C for 2 hours to precipitate proteins. After centrifugation at 25,000 g for 15 minutes at 4 °C, the supernatant containing the metabolites was transferred to a new tube and dried under vacuum. The dried residue was reconstituted in 180 μL of methanol:water (1:1, v/v), vortexed for 10 minutes, and centrifuged again at 25,000 g for 15 minutes at 4 °C. The final supernatant was collected for ultra-performance liquid chromatography–mass spectrometry (UPLC-MS) analysis.

### UPLC-MS detection of metabolites in serum

The UPLC-MS detection of metabolites in serum was performed by BGI Co., Ltd. (Shenzhen, China), using a Waters 2777c UPLC (Waters Corp., USA) in series with a Q Exactive HF high resolution mass spectrometer (Thermo Fisher Scientific, USA). Chromatographic separation, Primary and secondary MS data acquisition were performed as previously described (31).

### Analysis of metabolites

Partial least squares-discriminant analysis (PLSDA) (32) and orthogonal partial least squares discriminant analysis (OPLSDA) (33) between the comparative GBS group and non-GBS group were performed as previously described. The variable importance in projection (VIP) method was used to measure the influence intensity and interpretation ability for each metabolite. Using a VIP score of greater than 1 is the typical method for selecting relevant variables (34, 35), and this method was used to identify potentially important metabolites in GBS. Classification and functional annotation analysis of the identified metabolites was performed using the KEGG (36) and HMDB databases (37).

### Metagenomics sequencing and analysis

Metagenomic extraction from GBS stool samples and metagenomics PE150 sequencing based on the MGISEQ-2000 platform were performed at the BGI Co., Ltd. (Shenzhen, China). Raw reads were quality−filtered using SOAPnuke (38) to remove reads containing ≥10% ambiguous bases (N bases), reads with sequencing adapters, and reads with >50% low−quality bases (Phred score <20). Host contamination was removed by aligning clean reads to the human reference genome (GRCh38) using Bowtie2 (39). The high−quality, host−depleted reads were assembled into contigs using MEGAHIT (k−mer based, contigs <300 bp filtered) (40). Ab initio prediction of metagenomic genes from GBS stool samples was conducted using MetaGeneMark (41), followed by redundancy removal using CD−HIT (identity threshold 95%, coverage 90%). Relative abundance of each gene was quantified using Salmon (42) and normalized as transcripts per million (TPM). For species annotation, reads were classified using Kraken2 (43) against a custom database (NCBI NT and UHGG) and relative species abundance was estimated using Bracken2. Functional gene annotations were analyzed by eggNOG (44), KEGG (36), BacMet (45), COG (46), CAZy (47), CARD (48) and SwissProt (49). Species composition and functional composition differences between GBS and non-GBS samples were analyzed by NMDS (50). Analysis of similarities (ANOSIM) was performed as previously described (51). Alpha diversity and beta diversity were conducted as previously described to compare microbial community structure between GBS and Non-GBS control groups (52). Inter−group differential species were identified using LEfSe (Linear discriminant analysis Effect Size) analysis (53). Functional pathway enrichment was assessed using the Reporter score method based on KEGG orthologs (54).

### Correlational analysis

Pearson’s correlation analysis was conducted to assess the associations between the relative abundances of gut microbial species and the normalized peak intensities of serum metabolites. Only microbial species and metabolites that were significantly different between the GBS and control (non-GBS) groups were included in the analysis. For each microbe–metabolite pair, Pearson correlation coefficients (r) and corresponding p-values were calculated. To account for multiple comparisons, p-values were adjusted using the Benjamini–Hochberg false discovery rate (FDR) correction method, and adjusted q-values < 0.05 were considered statistically significant. Only correlations with an absolute correlation coefficient (|r|) > 0.5 and q < 0.05 were retained for downstream analysis and reporting.

### Isolation of intestinal microbial strains

Seven different media (LB agar, MRS agar containing 2% CaCO_3_, R2A agar, SS agar, Brain-heart infusion medium, Columbia Blood Agar Plate and Skirrow Medium) were used to isolate different intestinal microbial strains. GBS stool samples were serially diluted 10-fold with sterilized water, separately plated onto the above seven media, and incubated overnight at 37 °C to obtain single colonies. Then, a selected single colony was streaked three consecutive times on the corresponding solid media to obtain a pure culture. The 16S rDNA gene of a pure culture was amplified by PCR using the universal primers 27F (5’-AGAGTTTGATCCTGGCTCAG-3’) and 1492R (5’-GGTTACCTTGTTACGACTT-3’), and then PCR products were purified using PCR Clean Up Kit (Beyotime, China) and sequenced at BioSune Co., Ltd. (Shanghai, China). The 16S rDNA sequence was analysed using BLAST (https://blast.ncbi.nlm.nih.gov/Blast.cgi) for preliminary identification.

## Results

### GBS subjects have an altered gut microbiota, including enrichment of *Ligilactobacillus salivarius*, *Klebsiella pneumonia* and *Methanobrevibacter smithii*

**Figure 1:**
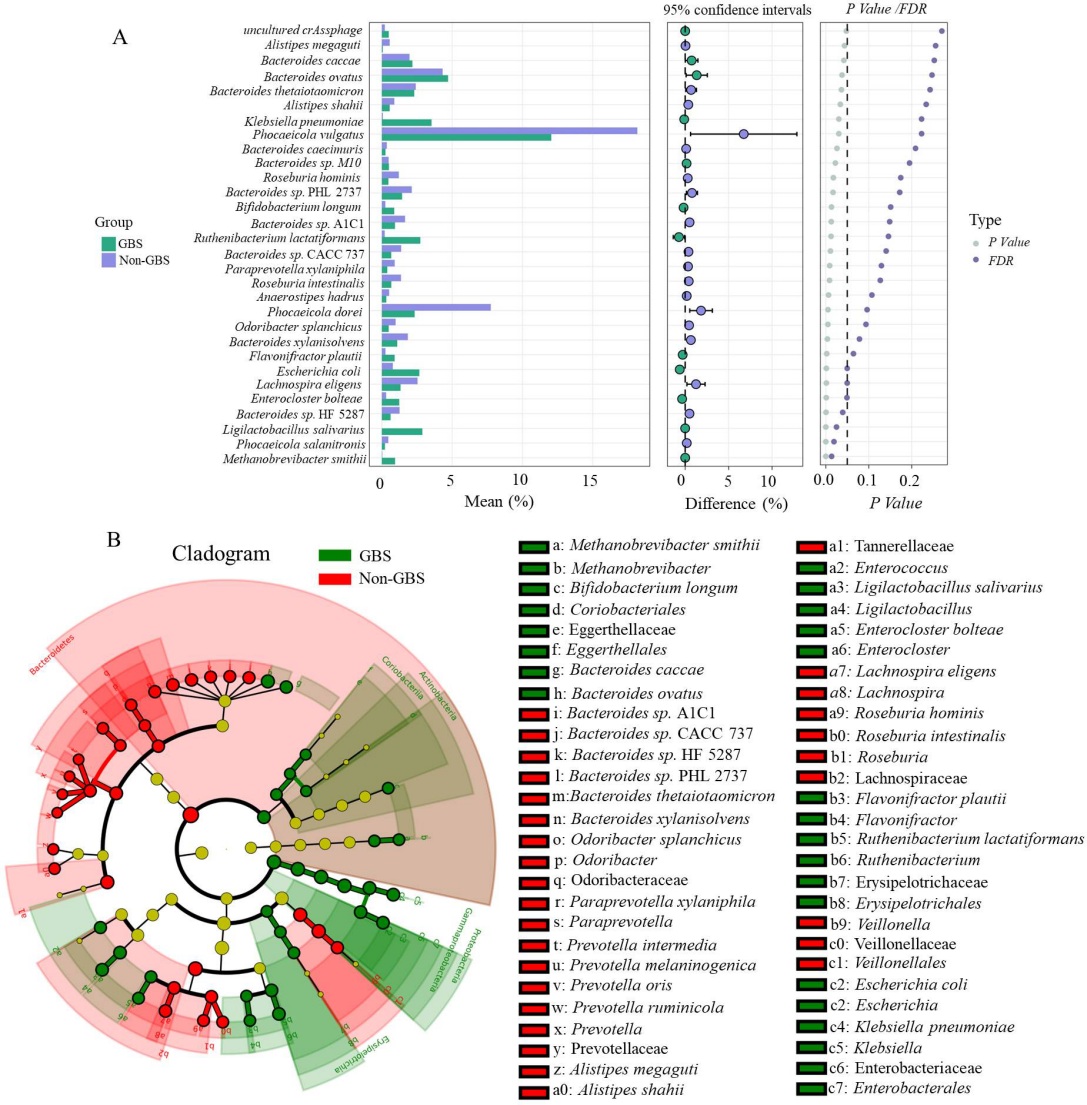
Metagenomic species analysis of stool samples from GBS and non-GBS groups. **(A)** Microbiotic mean abundance of gut microbiota in GBS and non-GBS subjects at the species level. **(B)** Cladogram indicating the phylogenetic distribution of microbiota that correlate with the GBS or the control non-GBS groups.

The microbial populations in 30 qualified stool samples from GBS subjects and 30 qualified stool samples from non-GBS subjects were compared by metagenomics sequencing. The rarefaction curve of GBS and Non-GBS group reached a plateau (**Supplementary Figure 1**), indicating that the sampling quantities for the GBS and non-GBS groups were reasonable and sufficient. Both PLSDA and ANOSIM statistical analyses showed that the inter-group difference between GBS and non-GBS group was greater than the intra-group difference at the family level, genus level, and species level (**Supplementary Figure 2**), indicating distinct differences in the gut microbiota between the two groups of subjects. At the species level, the most significantly enriched species in the GBS group were *Ligilactobacillus salivarius*, *Klebsiella pneumonia*, *Methanobrevibacter smithii*, *Ruthenibacterium lactatiformans*, *Enterocloster bolteae* and *Escherichia coli*, while *Bacteroides* sp. CACC 737, *Bacteroides* sp. HF-5287, *Roseburia hominis*, *Paraprevotella xylaniphila* and *Odoribacter splanchnicus* were decreased significantly in GBS (**Figure 1A**). Based on the mean relative abundance shown in **Figure 1A**, the calculated fold changes (GBS/Non-GBS) for these species were 1836−fold, 48.74−fold, 26.82−fold, 13.50−fold, 4.01−fold, 3.4−fold (for the enriched species) and 0.48−fold, 0.49−fold, 0.38−fold, 0.42−fold, 0.49−fold (for the decreased species), respectively. LEfSe analysis also indicated significant differences in the microbiomes between the two groups, with *M. smithii*, *L. salivarius*, *K. pneumonia*, *E. coli* and *R. lactatiformans* among the most notable microbes in the GBS group (**Figure 1B**).

At the genus level, *Ligilactobacillus*, *Klebsiella*, *Methanobrevibacter*, *Escherichia*, *Enterocloster*, *Paraprevotella*, *Roseburia* and *Odoribacter* had changes in levels consistent with those at the species level in the GBS group, while *Enterococcus* and *Ruthenibacteriu* were also enriched (**Supplementary Figure 3A**), although no enrichment was detected in the corresponding species. Based on the mean relative abundance shown in **Supplementary Figure 3A**, the calculated fold changes (GBS/Non-GBS) for these genera were 162−fold, 35.6−fold, 26.6−fold, 3.4−fold, 2.9−fold, 0.42−fold, 0.44−fold, 0.50−fold, 25.3-fold, 13.5-fold, respectively. In addition, at the family level, Enterobacteriaceae was significantly enriched (**Supplementary Figure 3B**), and this family includes some notable pathogens, such as *Klebsiella pneumonia*, which was also enriched in the GBS group. KEGG analysis of the metagenome data also revealed that genes belonging to pathways associated with bacterial infection were enriched in GBS (**Supplementary Figure 4**), suggesting that GBS is associated with bacterial infection.

### Pathogens isolated from GBS stool samples

Metagenomic data revealed that pathogens such as *Klebsiella pneumonia* and *Escherichia coli* were abundant in GBS subjects, indicating that GBS may be related with infection of these pathogens. Seven different media were further used to isolate the potential pathogens from GBS stool samples. As shown in **Table 1**, *Escherichia coli* and *Klebsiella pneumoniae* were isolated using LB agar, R2A agar, SS agar, Brain-heart infusion medium. *Streptococcus lutetienis* and *Streptococcus lutetienis* were isolated using MRS agar containing 2% CaCO_3_ and Brain-heart infusion medium. *Enterobacter sakazakii* was isolated using R2A agar. *Campylobacter jejuni* has been reported to be associated with GBS (10). However, in this study, no *Campylobacter jejuni* were isolated using the special separation Skirrow Medium.

**Table 1:** Gut microbes isolated from GBS subjects.

Culture medium	Isolates
LB agar	*Escherichia coli*, *Klebsiella pneumoniae*
MRS agar containing 2% CaCO_3_	*Streptococcus lutetienis*
R2A agar	*Escherichia coli*, *Klebsiella pneumoniae*, *Enterobacter sakazakii*
SS agar	*Klebsiella pneumoniae*, *Escherichia coli*
Brain-heart infusion medium	*Klebsiella pneumoniae, Escherichia coli, Streptococcus lutetienis*
Columbia Blood Agar Plate	*Escherichia coli*
Skirrow Medium	no *Campylobacter jejuni* isolates

### Metabolites in GBS serum show significant changes

**Figure 2:**
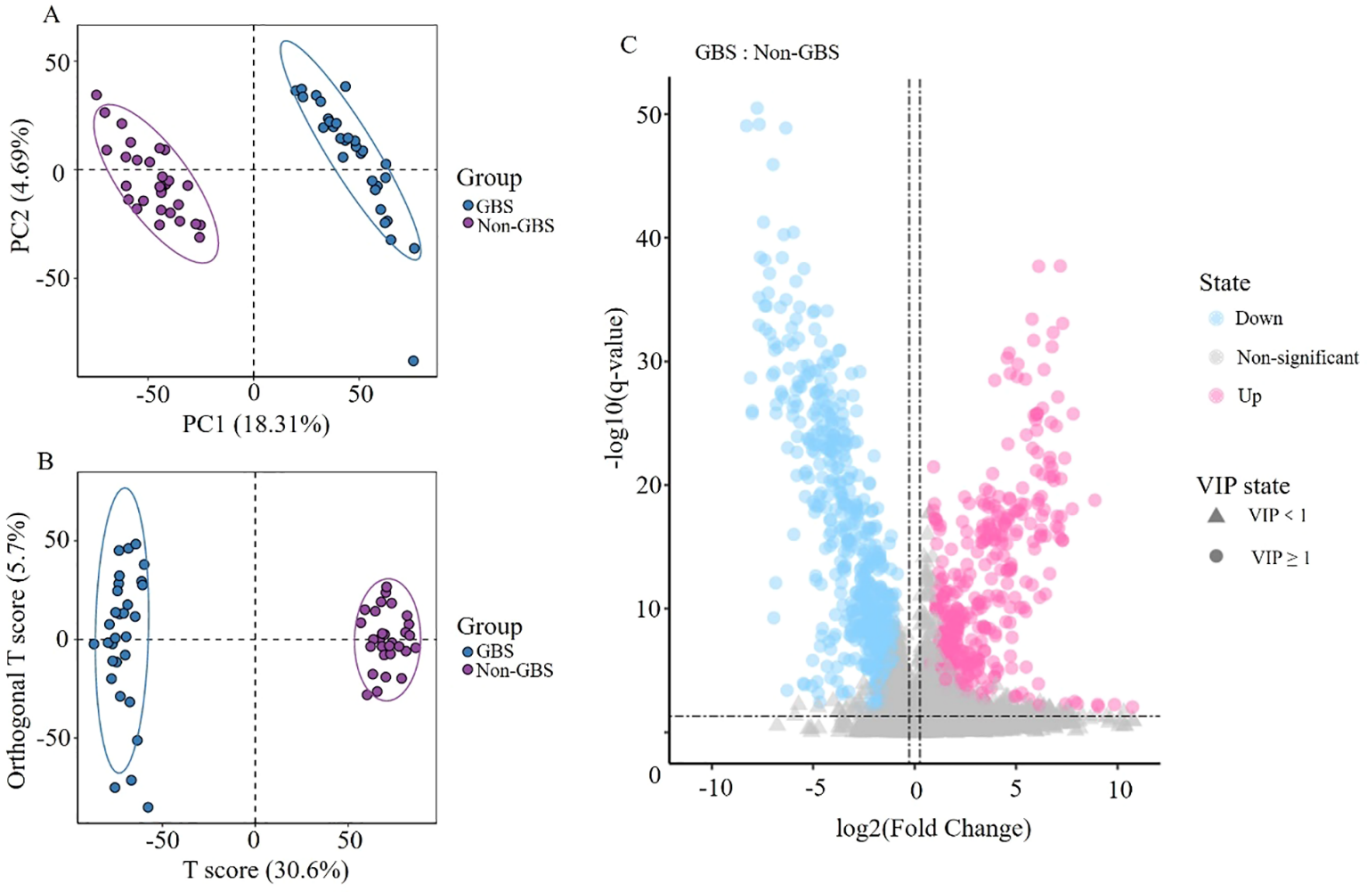
Multivariate statistical analysis and screening of differential metabolites. **(A)** PLSDA score map for metabolites. The numbers in parentheses are the scores for the principal component, which represents the percentage of the explanation on overall variance of the specific principal component. **(B)** OPLSDA score map for metabolites. **(C)** Volcano map of differential metabolites showing 672 metabolites decreased and 356 metabolites increased in GBS subjects.

Thirty serum samples from GBS subjects and 30 serum samples from healthy non-GBS subjects were analyzed by LC-MS/MS, and 4060 metabolites were identified in both groups, of which 1028 were characterized as differential metabolites. PLSDA (**Figure 2A**) and OPLSDA (**Figure 2B**) were performed to observe the distribution and separation trend of the GBS and non-GBS groups of samples, and both the PLSDA and OPLSDA models showed that the major patterns of serum metabolites were largely distinct for GBS patients versus non-GBS controls. Further statistical analysis of the 1028 differential metabolites revealed that 672 metabolites were decreased and 356 metabolites were increased in the GBS group (**Figure 2C**), indicating broad metabolic shifts in GBS subjects versus non-GBS controls.

### Enrichment in gamma-aminobutyric acid in GBS

**Figure 3:**
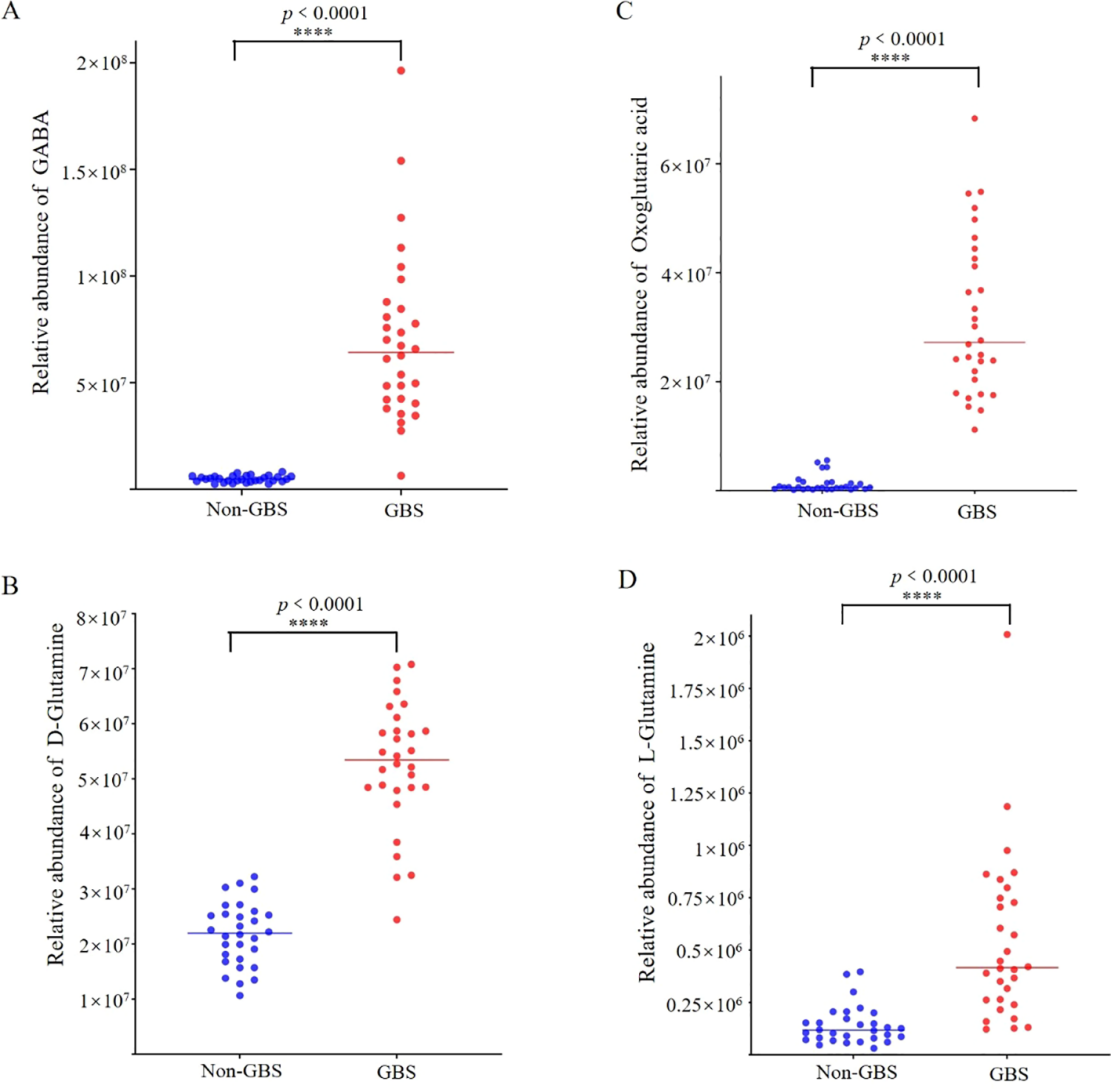
Significant enrichment of GABA and related metabolites in serum of GBS subjects. Relative abundance of **(A)** GABA, **(B)** D-glutamine, **(C)** oxoglutaric acid and **(D)** L-glutamine in serum of GBS vs non-GBS subjects. ****p < 0.0001.

Gamma-aminobutyric acid (GABA) is an inhibitory neurotransmitter found in the brain (55). Our analysis of the enriched serum metabolites revealed that GABA was increased significantly (~14.3 fold) in GBS subjects and that oxoglutaric acid (~25.35-fold), L-glutamine (~3.8-fold) and D-glutamine (~2.4-fold), which are associated with GABA metabolism, were also increased significantly (**Figure 3**). Quantification and comparison of GBS serum metabolites revealed that the GABA and D-glutamine metabolic pathways were also significantly enriched in GBS patients (**Supplementary Table 2**), consistent with the increased GABA and glutamine levels in GBS serum.

### Increased GABA levels correlate with enrichment of *Ligilactobacillus salivarius, Methanobrevibacter smithii and Enterococcus* species

GABA is produced by gut microbes, such as *Enterococcus avium*, *Pseudomonas* sp., *Streptococcus thermophilus*, *Lactiplantibacillus plantarum*, *Lactobacillus brevis* and *Bifidobacterium dentium* (56–59), and *Methanobrevibacter smithii* plays a key role in energy harvesting and carbon metabolism in gut (60). As indicated above, there were striking differences in the serum GABA of GBS subjects and non-GBS subjects. Additionally, metagenome data revealed that *Enterococcus* species, which are reported to be associated with the production of GABA (61, 62), were increased in GBS subjects versus non-GBS subjects. Since *Ligilactobacillus salivarius* and *Methanobrevibacter smithii* levels were also particularly high in GBS subjects, our results indicate that *Enterococcus* species, *L. salivarius* and *M. smithii* may be associated with GABA production in GBS subjects.

**Figure 4:**
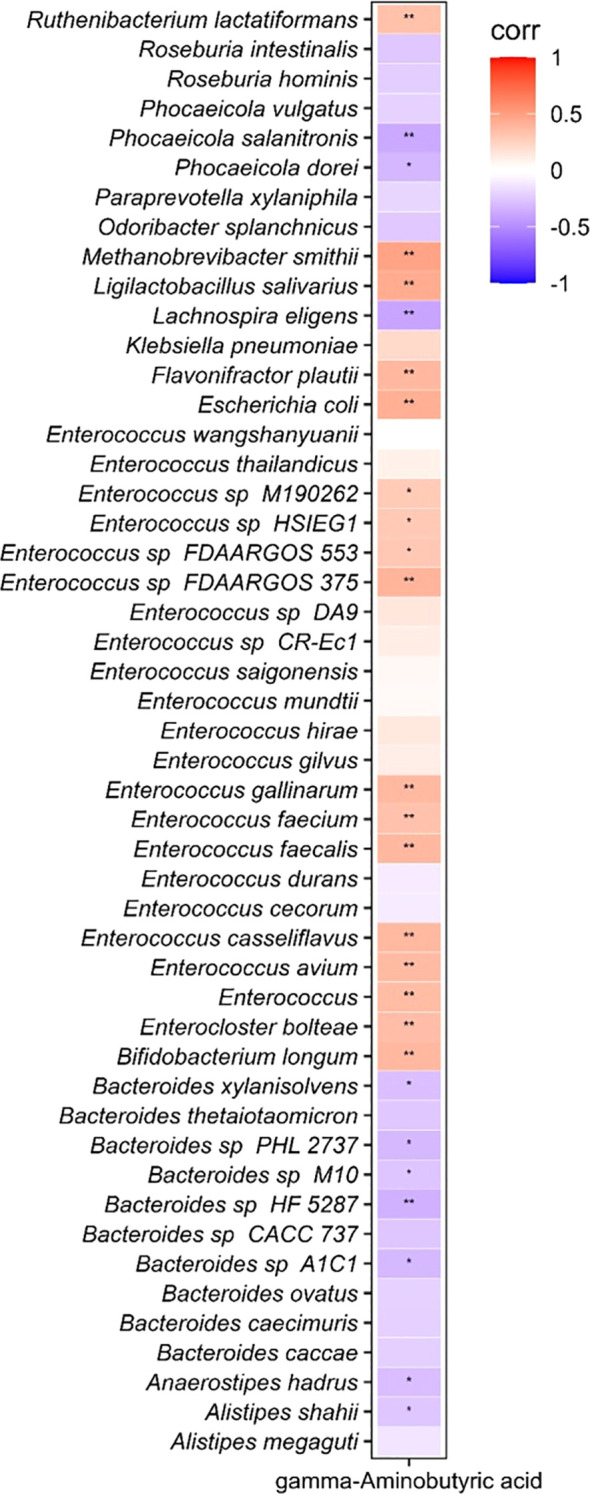
Relationship between GABA levels and the corresponding relative abundance of 49 selected microbial species. The colored scale shows the relative abundances and correlation values. *p < 0.05, **p < 0.01.

Pearson’s correlation analyses found that the increased GABA levels in the serum of GBS subjects were significantly and positively correlated with the abundance of *Enterococcus* species (such as *Enterococcus* sp. FDAARGOS 375, *Enterococcus gallinarum*, *Enterococcus faecalis*, *Enterococcus faecium*, *Enterococcus casseliflavus* and *Enterococcus avium*); *Ligilactobacillus salivarius*; *Methanobrevibacter smithii*, *Ruthenibacterium lactatiformans*; *Flavonifractor plautii* and *Escherichia coli* (**Figure 4**). In addition, function analysis of the stool metagenome data revealed that the GABA biosynthesis pathways of prokaryotes were enriched and that the GABA-relevant metabolic pathway in which arginine is converted into glutamate was also enriched in gut microbiota (**Supplementary Table 3**), indicating that the abnormal GABA levels in GBS serum might be associated with the altered composition of gut microbiota.

### Microbial-derived secondary cholic acids are decreased in GBS

**Figure 5:**
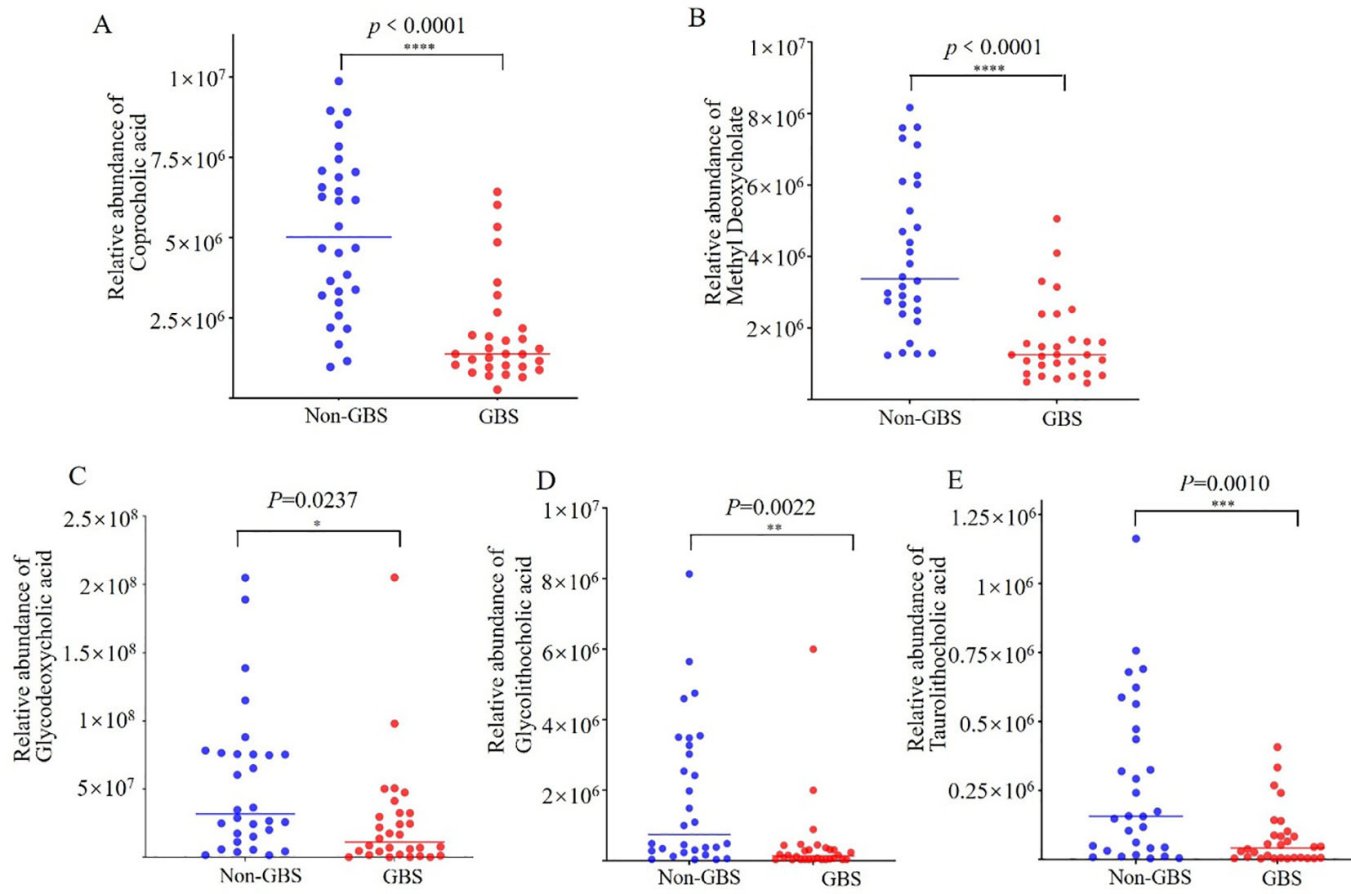
Significantly decreased levels of secondary cholic acids in GBS subjects. Relative abundance of **(A)** coprocholic acid, **(B)** methyl deoxycholate, **(C)** glycodeoxycholic acid, **(D)** glycolithocholic acid and **(E)** taurolithocholic acid in the serum of GBS vs non-GBS subjects. *p < 0.05, **p < 0.01, ***p < 0.001, ****p < 0.0001.

Secondary cholic acids are produced by gut microbes, such as *Bacteroides*, *Clostridium*, *Lactobacillus* and *Ruminococcus*, via transformation of host-derived primary cholic acids (63). Analysis of the serum metabolites revealed that microbial-derived secondary cholic acids were changed significantly in the GBS group versus non-GBS controls, with all of these secondary cholic acids showing decreased levels in GBS serum, including methyl deoxycholate (~0.39-fold), glycodeoxycholic acid (~0.47-fold), glycolithocholic acid (~0.24-fold), taurolithocholic acid (~0.28-fold) and coprocholic acid (~0.39-fold) (**Figure 5**); in contrast, all primary cholic acids remained unchanged in GBS serum versus non-GBS serum.

### Gut microbiota disturbance affects secondary cholic acid levels in GBS

**Figure 6:**
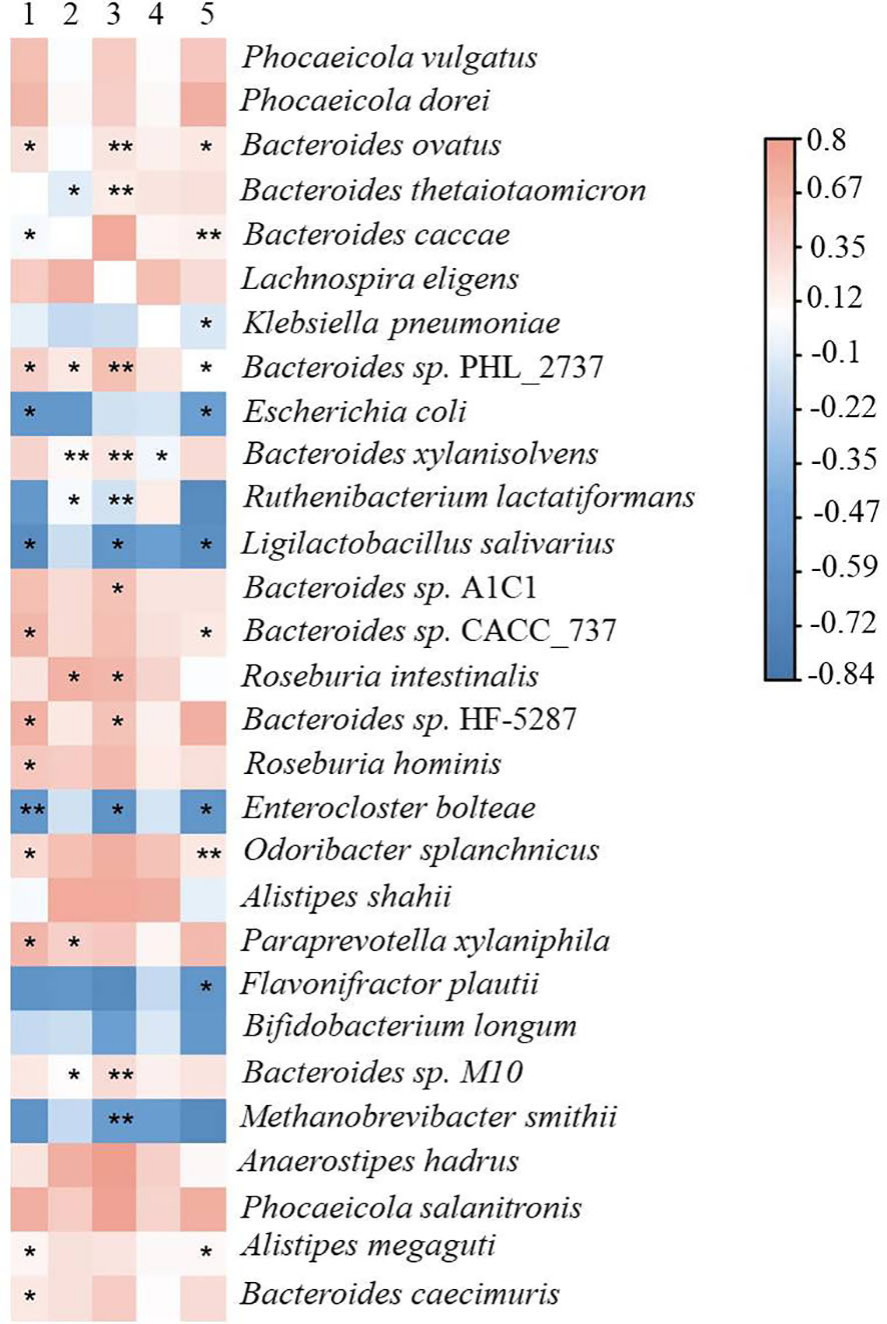
Relationship between levels of secondary cholic acids and corresponding relative abundance of 29 selected microbial species. Column 1, methyl deoxycholate; column 2, glycodeoxycholic acid; column 3, glycolithocholic acid; column 4, taurolithocholic acid; and column 5, coprocholic acid. The colored scale shows the relative abundances and correlation values. *p < 0.05, **p < 0.01.

As noted above, the secondary cholic acids had significantly lower levels in the serum of GBS subjects versus non-GBS subjects (**Figure 5**). Additionally, metagenome data revealed that gut microbes such as *Bacteroides* that are associated with the transformation of secondary cholic acids were decreased in GBS subjects versus non-GBS subjects (**Figure 1A**), indicating that the secondary cholic acid metabolic pathways of microorganisms were abnormal in GBS. Correlation analysis revealed that the changes in secondary cholic acids were positively correlated with the levels of *Bacteroides* species and *Roseburia* species (**Figure 6**), which were all decreased in the gut microbiota in the GBS group. These results suggested that changes in the secondary cholic acids might be associated with the altered composition of gut microbiota of the GBS subjects.

## Discussion

Both GBS and CIDP are rare, immune-mediated diseases of the complex peripheral nervous system (64, 65). Although the pathogenic factors that activate the immune system in these diseases are unknown, prodromal infection may be the cause (6, 7). In a previous report, we found that CIDP was associated with gut microbiota alterations affecting bile acid and arachidonic acid metabolism (27). In this study, we focused on GBS and identified significant disturbances in GABA and secondary bile acid metabolism. While some gut microbes (e.g., *Klebsiella pneumoniae*, *Escherichia coli*, and *Ligilactobacillus salivarius*) were enriched in both GBS and CIDP (**Supplementary Table 4**), other changes were disease−specific. For instance, *Methanobrevibacter smithii* was markedly enriched only in GBS (26.82−fold) but decreased in CIDP (**Supplementary Table 5**), *Megamonas funiformis* and *Phascolarctobacterium faecium* were only enriched in CIDP and remained unchanged in GBS (**Supplementary Table 6**), suggesting that disturbances in gut microbe levels play different and potentially important roles in the development and progression of GBS and CIDP.

The most common infectious pathogens that trigger GBS include *Campylobacter jejuni*, *Mycoplasma pneumoniae* and cytomegalovirus (10, 66, 67); surprisingly, these pathogens were not detected in the stool samples from GBS subjects in this study. However, the opportunistic pathogen *Klebsiella pneumonia* and *Escherichia coli* were highly enriched (**Figure 1**) and isolated (**Table 1**) in GBS subjects versus non-GBS subjects. Additionally, function analysis of stool metagenome data revealed that O-antigen repeat unit biosynthesis, O-antigen nucleotide sugar biosynthesis and lipopolysaccharide biosynthesis, which are associated with bacterial infection (68, 69), were enriched (**Supplementary Table 7**), and KEGG analysis revealed that infectious disease pathways in GBS subjects were also enriched (**Supplementary Figure 4**), further suggesting that bacterial infections, potentially including *Klebsiella pneumonia* and *Escherichia coli* infections, are associated with GBS. Notably, these two pathogens have been reported to be associated with neurological diseases (70–73).

**Figure 7:**
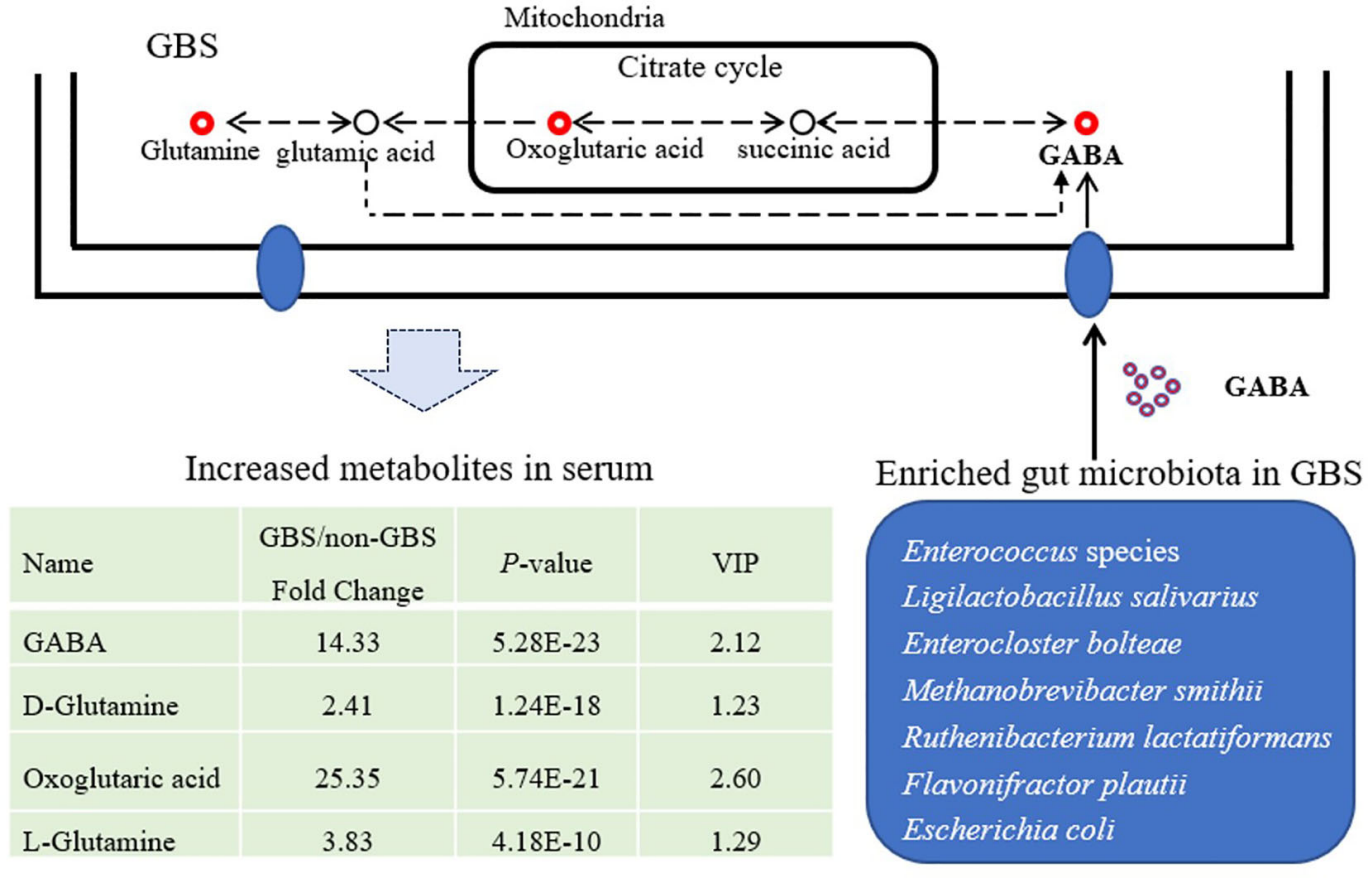
Relationship between GABA levels and gut microbiota in GBS subjects. GABA-producing microbes, such as *Enterococcus* species, *Enterocloster bolteae*, *Ligilactobacillus salivarius* and *Methanobrevibacter smithii*, were enriched in the gut of GBS subjects, and the GABA biosynthesis pathway was also enriched in the gut microbes of GBS subjects. Therefore, gut microbes may be the source of the increased GABA levels found in the serum of GBS subjects.

Our finding of elevated serum GABA in GBS has not been previously reported. A recent study identified cytokines such as IL−9 as potential biomarkers in GBS (2), while GABA has not been studied. Previous studies found that GABA could be produced by gut microbes, such as *Enterococcus avium*, *Lactiplantibacillus plantarum*, *Lactobacillus brevis* and *Bifidobacterium dentium* (56–59). In this study, the increased GABA in GBS subjects was positively correlated with *Enterococcus* species, *Ligilactobacillus salivarius* and *Methanobrevibacter smithii*, suggesting that gut microbes are involved in the transformation of GABA in GBS (**Figure 7**). Furthermore, *Methanobrevibacter smithii* had a particularly striking enrichment in GBS (26.82-fold), and the increased GABA level in the serum of GBS subjects was significantly and positively correlated with this *Methanobrevibacter smithii* enrichment (**Figure 7**). *Methanobrevibacter smithii* is strictly anaerobic and is almost always found in the human digestive tract, although in very low abundance (74); therefore the specific enrichment of this species in the GBS subjects is intriguing. These findings suggest that *Methanobrevibacter smithii* may be involved in GBS disease progression through the production of metabolites such as GABA.

## Conclusion

This study revealed that GABA metabolism and secondary cholic acid metabolism were perturbed in GBS subjects versus non-GBS controls and that serum GABA increased significantly, while secondary cholic acids decreased significantly, in GBS subjects. Enrichment of the GABA biosynthesis pathway in gut microbes correlated with a greater abundance of *Enterococcus* species, *Enterocloster bolteae*, *Ligilactobacillus salivarius* and *Methanobrevibacter smithii*, whereas the decrease in secondary cholic acid metabolism pathways in gut microbes was associated with altered levels of *Bacteroides* species and *Roseburia* species. Our findings suggest that GBS may be related to alterations in the gut microbiota, including changes in the levels of gut microbes that could impact GABA and secondary cholic acid metabolism, and to the presence of potential pathogens such as *Klebsiella pneumoniae* and *Escherichia coli*. These findings also indicate that the normal microbiota-host interactions are disturbed with GBS. Finally, GABA may be a promising biomarker for the diagnosis of GBS, and modulation of the gut microbiota could potentially impact the clinical course of GBS.

## Data Availability

Metagenome data were deposited to NCBI with SRA data: PRJNA1121414. Metabolomics data have been deposited to the EMBL-EBI MetaboLights database with the identifier MTBLS10392.
