# Supplementary material for: Altered GABA and secondary bile acids in Guillain-Barré syndrome: association with gut dysbiosis
Source: Front Immunol. 2026 Jun 10;17:1849216. doi: 10.3389/fimmu.2026.1849216 (PMC13290612; doi:10.3389/fimmu.2026.1849216)
Supplement: Supplementary file 1 [file DataSheet1.doc]

**Supplemental material**

**Altered GABA and secondary bile acids in Guillain-Barré syndrome: association with gut dysbiosis**

Jiafang Fu^1,2,3^, Jingli Shan^4^, Hua Xu^1^, Zhiwei Zhu^5^, Pengshuo Yang^1,2,3^, Qinzhou Wang^4^*, Jinxiang Han^1,2,3^* and Guangxiang Cao^1,2,3^*

^1^Department of Neurology, The First Affiliated Hospital of Shandong First Medical University & Shandong Provincial Qianfoshan Hospital, Jinan 250014, China;

^2^Biomedical Sciences College and Shandong Medicinal Biotechnology Centre, Shandong First Medical University and Shandong Academy of Medical Sciences, Jinan 250117, China;

^3^NHC Key Laboratory of Biotechnology Drugs, Shandong Academy of Medical Sciences, Jinan 250117, China;

^4^Research Institute of Neuromuscular and Neurodegenerative Diseases and Department of Neurology, Qilu Hospital, Cheeloo College of Medicine, Shandong University, Jinan 250012, China;

^5^ Shandong Institute of Industrial Technology for Health Sciences and Precision Medicine, Jinan 250101, China

Address correspondence to Qinzhou Wang, Shandong University, Cheeloo College of Medicine, Jinan 250012, China. E-mail: Qinzhouwang@163.com;

Jinxiang Han, Shandong First Medical University & Shandong Academy of Medical Sciences, Jinan 250117, China. E-mail: jxhan@sdfmu.edu.cn;

and Guangxiang Cao, Shandong First Medical University & Shandong Academy of Medical Sciences, Jinan 250117, China. E-mail: caoguangxiang@sdfmu.edu.cn.

**Table S1 Cohort of GBS patients and healthy controls enrolled in this study.**

| Subject ID | Age | Gender | Subject | Vaccination prior to onset | Infection prior to onset | Taking antibiotics prior to onset | |
| --- | --- | --- | --- | --- | --- | --- | --- |
| D1 | 55 | female | GBS | No | Diarrhea | No |  |
| D7 | 22 | male | GBS | No | No | No |  |
| D10 | 65 | female | GBS | No | No | No |  |
| D11 | 29 | male | GBS | No | No | No |  |
| D12 | 36 | male | GBS | No | No | No |  |
| D14 | 47 | female | GBS | No | No | No |  |
| D15 | 20 | male | GBS | No | Fever/diarrhea | No |  |
| D16 | 53 | male | GBS | No | Upper respiratory tract infection | No |  |
| D17 | 35 | male | GBS | No | Fever/diarrhea | No |  |
| D18 | 51 | male | GBS | No | No | No |  |
| D21 | 27 | female | GBS | COVID-19 | Fever | Ibuprofen |  |
| D24 | 65 | male | GBS | No | Fever | No |  |
| D31 | 71 | male | GBS | No | Diarrhea | No |  |
| D32 | 42 | male | GBS | No | Diarrhea | No |  |
| D34 | 62 | male | GBS | No | No | No |  |
| D35 | 48 | female | GBS | No | Upper respiratory tract infection | No |  |
| D36 | 52 | female | GBS | No | Upper respiratory tract infection | No |  |
| D37 | 60 | female | GBS | No | Fever | No |  |
| D38 | 65 | male | GBS | No | Diarrhea | No |  |
| D39 | 58 | male | GBS | No | No | No |  |
| D40 | 55 | male | GBS | No | Upper respiratory tract infection | No |  |
| D42 | 38 | male | GBS | No | No | No |  |
| D43 | 55 | male | GBS | No | No | No |  |
| D45 | 54 | female | GBS | No | No | No |  |
| D46 | 32 | female | GBS | No | No | No |  |
| D47 | 30 | female | GBS | No | No | No |  |
| D48 | 21 | female | GBS | No | No | No |  |
| D49 | 31 | male | GBS | No | No | No |  |
| D50 | 31 | male | GBS | No | No | No |  |
| D51 | 68 | male | GBS | No | No | No |  |
| K1 | 35 | male | non-GBS | No | No | No |  |
| K2 | 66 | male | non-GBS | No | No | No |  |
| K3 | 33 | male | non-GBS | No | No | No |  |
| K4 | 48 | female | non-GBS | No | No | No |  |
| K5 | 40 | female | non-GBS | No | No | No |  |
| K6 | 23 | male | non-GBS | No | No | No |  |
| K7 | 61 | male | non-GBS | No | No | No |  |
| K8 | 20 | female | non-GBS | No | No | No |  |
| K9 | 50 | male | non-GBS | No | No | No |  |
| K10 | 73 | male | non-GBS | No | No | No |  |
| K11 | 34 | male | non-GBS | No | No | No |  |
| K12 | 29 | male | non-GBS | No | No | No |  |
| K13 | 55 | female | non-GBS | No | No | No |  |
| K14 | 25 | female | non-GBS | No | No | No |  |
| K15 | 44 | male | non-GBS | No | No | No |  |
| K16 | 63 | female | non-GBS | No | No | No |  |
| K17 | 47 | female | non-GBS | No | No | No |  |
| K18 | 22 | male | non-GBS | No | No | No |  |
| K19 | 57 | female | non-GBS | No | No | No |  |
| K20 | 31 | female | non-GBS | No | No | No |  |
| K21 | 51 | male | non-GBS | No | No | No |  |
| K22 | 30 | male | non-GBS | No | No | No |  |
| K23 | 56 | male | non-GBS | No | No | No |  |
| K24 | 37 | male | non-GBS | No | No | No |  |
| K25 | 17 | male | non-GBS | No | No | No |  |
| K26 | 55 | female | non-GBS | No | No | No |  |
| K27 | 69 | male | non-GBS | No | No | No |  |
| K28 | 56 | male | non-GBS | No | No | No |  |
| K29 | 62 | male | non-GBS | No | No | No |  |
| K30 | 30 | female | non-GBS | No | No | No |  |
|  |  |  |  |  |  |  |  |

**Table S2 Enriched metabolites and their pathways identified in the metabolomics data of GBS subjects versus non-GBS subjects.**

| Pathway | Number of differentially abundant metabolites | Total number of metabolites in the pathway | Down | Up | P-value | Differentially abundant metabolites |
| --- | --- | --- | --- | --- | --- | --- |
| GABA metabolism | 3 | 9 | 0 | 3 | 8.22E-05 | Oxoglutaric acid; L-glutamine; GABA |
| D-glutamine metabolism | 3 | 13 | 0 | 3 | 2.72E-04 | Oxoglutaric acid; D-glutamine; L-glutamine |

**Table S3** GABA pathway report score analysis of stool metagenome data.

| Module_id | Map | Description | Report score |
| --- | --- | --- | --- |
| M00879 | map00330 Arginine and proline metabolism; map01100 Metabolic pathways | Arginine succinyltransferase pathway, arginine => glutamate | 3.50946023 |
| M00136 | map00330 Arginine and proline metabolism; map01100 Metabolic pathways | **GABA biosynthesis, prokaryotes, putrescine => GABA** | 2.272969365 |

**Table S4** Gut microbes with altered levels in both GBS and CIDP subjects.

| Species | GBS/non-GBS | *P-*value* | CIDP/non-CIDP | *P-*value |
| --- | --- | --- | --- | --- |
| *Klebsiella pneumoniae* | 48.74729784↑ | 0.03031743 | 96.73863416↑ | 0.000261689 |
| *Escherichia coli* | 3.417996601↑ | 0.00130167 | 2.388395752↑ | 0.00133681 |
| *Ligilactobacillus salivarius* | 1836.561999↑ | 0.000158461 | 95.84579588↑ | 0.000858486 |
| *Paraprevotella xylaniphila* | 0.422294603↓ | 0.00906876 | 0.565239576↓ | 0.047681709 |
| *Phocaeicola dorei* | 0.301008866↓ | 0.005084222 | 0.319337881↓ | 0.000908242 |
| *Bacteroides sp*. PHL 2737 | 0.676096256↓ | 0.01695488 | 0.534185858↓ | 0.006327964 |
| *Phocaeicola vulgatus* | 0.66301173↓ | 0.030317429 | 0.725710059↓ | 0.04604284 |
| *Phocaeicola salanitronis* | 0.475617685↓ | 5.97056E-05 | 0.88446655↓ | 0.038533344 |

**p*<0.05

**Table S5** Gut microbes that were increased only in GBS subjects.

| Species | GBS/non-GBS | *P-*value* | CIDP/non-CIDP | *P-*value |
| --- | --- | --- | --- | --- |
| *Ruthenibacterium lactatiformans* | 13.50018943↑ | 0.01221194 | 4.921526243 | 0.1967094 |
| *Enterocloster bolteae* | 4.069295596↑ | 0.001173758 | 1.480369425 | 0.4162595 |
| *Flavonifractor plautii* | 3.527481754↑ | 0.002156638 | 2.110550633 | 0.3918614 |
| *Bifidobacterium longum* | 3.600879403↑ | 0.013271805 | 3.570189701 | 0.0785966 |
| *Methanobrevibacter smithii* | 26.81665092↑ | 1.7115E-05 | 0.008694797 | 0.2791766 |

**p*<0.05.

**Table S6** Gut microbes that were increased only in CIDP subjects.

| Species | CIDP/non-CIDP | *P-*value* | GBS/non-GBS | *P-*value |
| --- | --- | --- | --- | --- |
| *Megamonas funiformis* | 3.016726357↑ | 0.0066264 | 1.226455618 | 0.9705161 |
| *Phascolarctobacterium faecium* | 3.244799642↑ | 0.0227444 | 1.502252839 | 0.8649937 |

**p*<0.05.

**Table S7** O-antigen pathway report score analysis of stool metagenome data.

| Pathway_id | Level 2 | Description | Report score |
| --- | --- | --- | --- |
| map00542 | Glycan biosynthesis and metabolism | O-antigen repeat unit biosynthesis | -2.476091208 |
| map00541 | Glycan biosynthesis and metabolism | O-antigen nucleotide sugar biosynthesis | -4.162517953 |
| map00540 | Glycan biosynthesis and metabolism | Lipopolysaccharide biosynthesis | -1.718372548 |


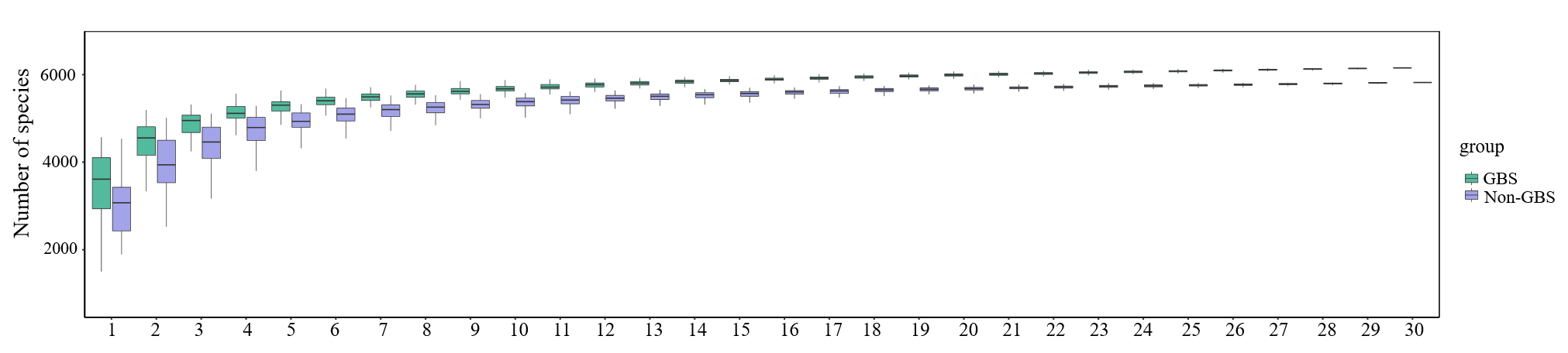


**Fig. S1** Rarefaction curve Boxplot. Abscissa represents for sample size while ordinate represents for number of species in sample. Diversity is limited when sample size is small, which is not reliable to represent for the entire microbiota structure. When rarefaction curve tends to be steady, it indicates that the sampling quantity is sufficient.


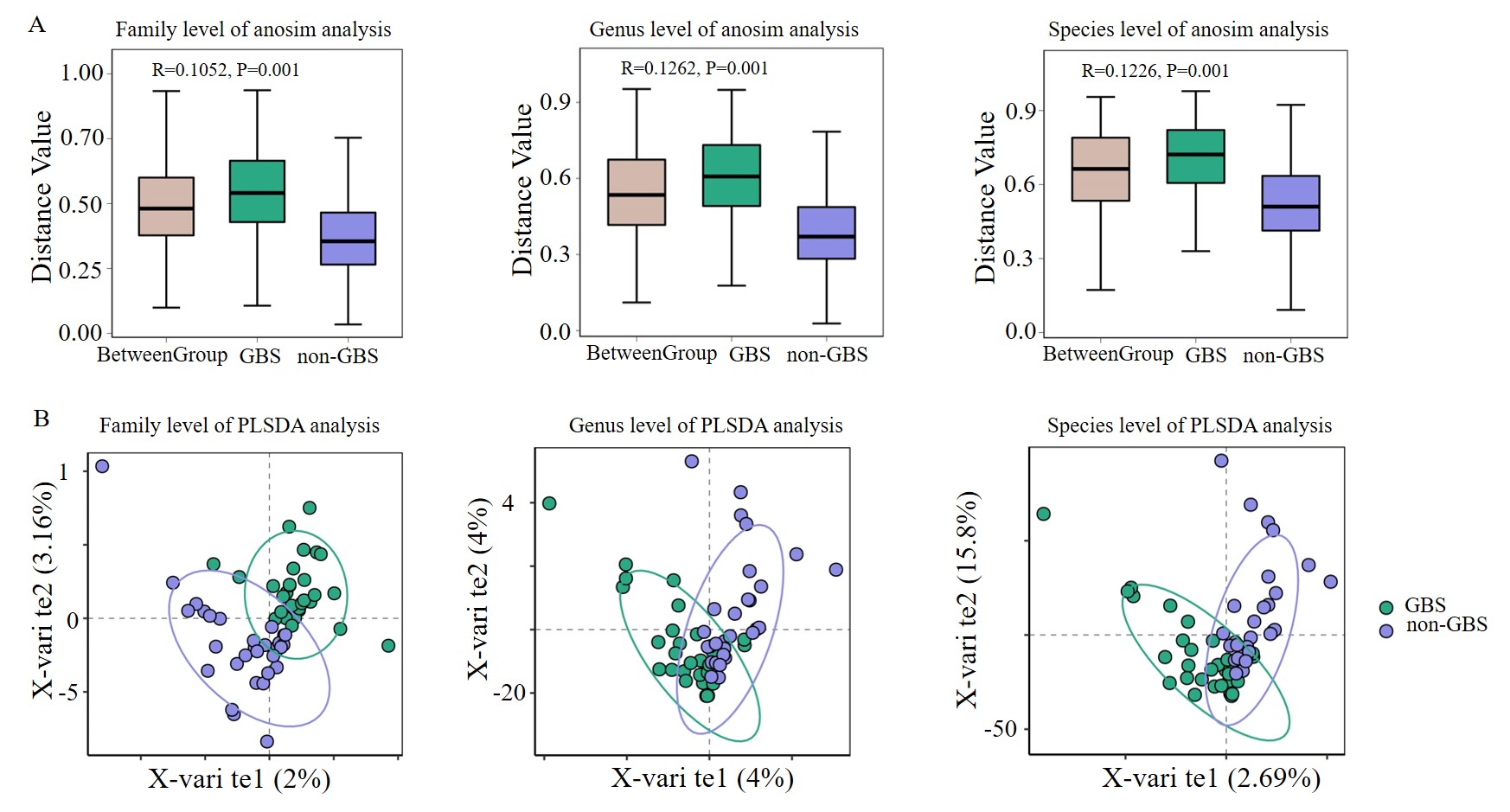


**Fig. S2** ANOSIM and PLSDA analysis. **A** ANOSIM analysis at the family level, genus level and species level. “Between Group” indicates the distance between GBS and non-GBS groups, and the remaining boxes indicate the distance within the corresponding group. **B** PLSDA analysis at the family level, genus level and species level.


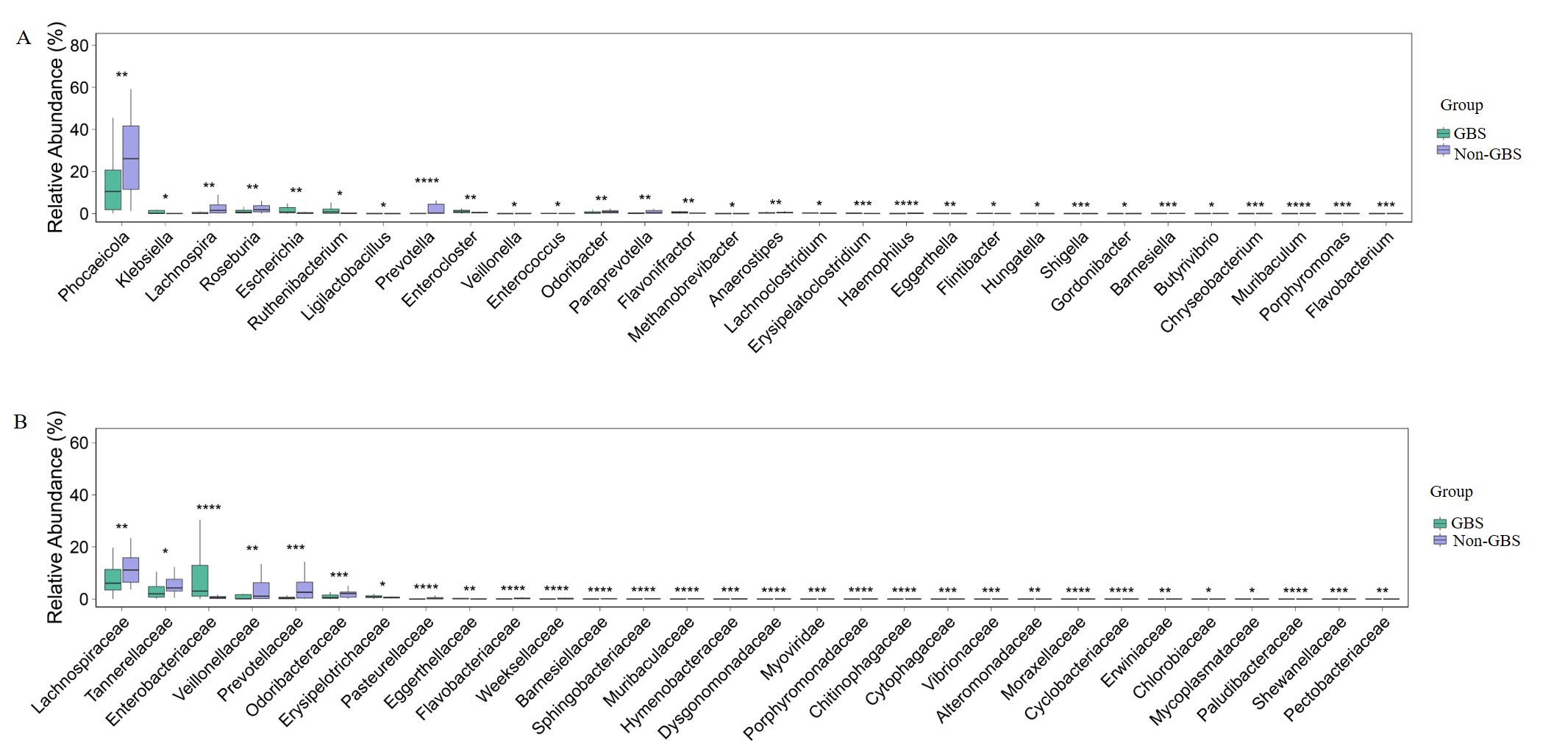


**Fig. S3** Taxonomic classifications and ratios of gut microbial flora in GBS and non-GBS groups. Microbiotic abundance is shown by taxonomy barplot at the genus level (A) and family level (B).


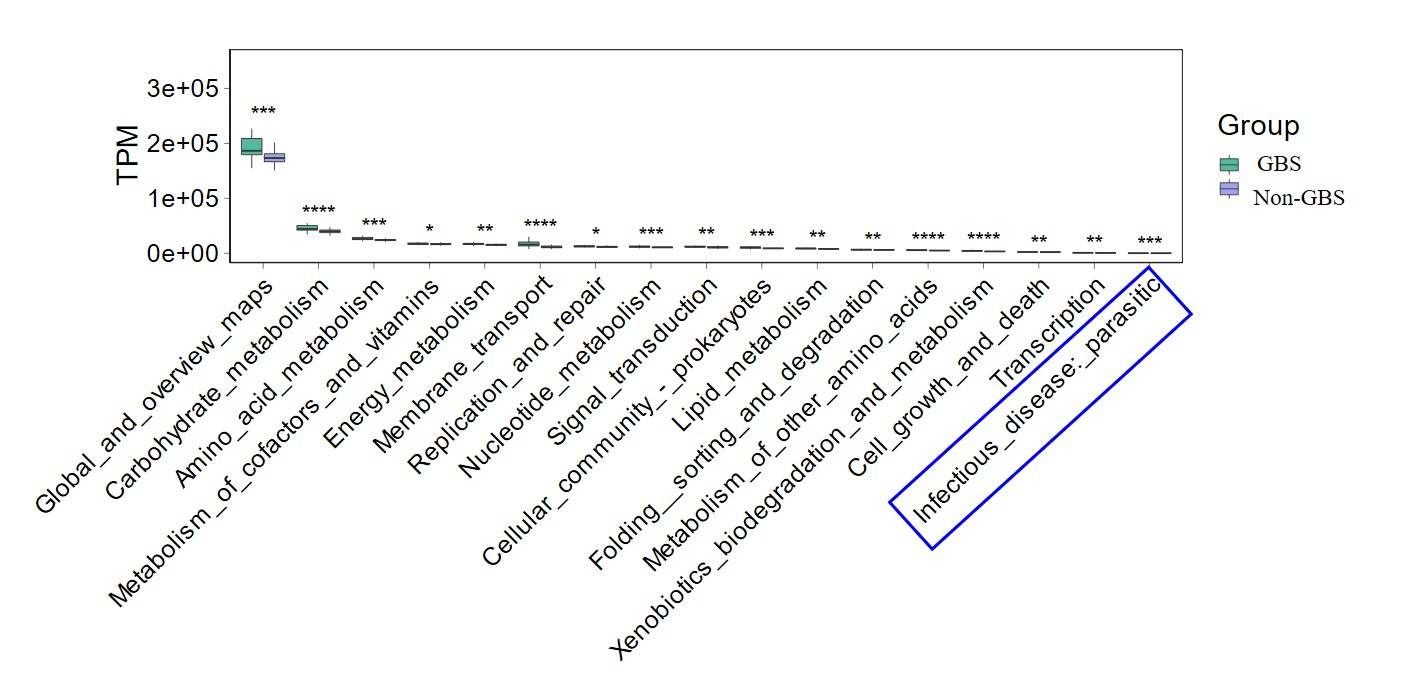


**Fig. S4** Level 2 KEGG pathway analysis of the stool metagenome of GBS and control subjects.
